# ‘Improving the odds for everybody’: Narrative and media in stem cell donor recruitment patient appeals, and the work to redress racial inequity

**DOI:** 10.1111/1467-9566.13505

**Published:** 2022-08-05

**Authors:** Ros Williams

**Affiliations:** ^1^ Department of Sociological Studies University of Sheffield Sheffield UK

**Keywords:** donation, ethnicity, health narratives, media, race, social media, stem cells

## Abstract

Stem cell registries, which provide cells for transplants in blood malignancy treatment, recruit donors partly through mobilising narrative. This is often via appeals from patients without matching donors who seek to encourage registrations from people who might go on to be their own, or somebody else’s, donor. Registries have also historically underserved racially minoritised communities, who are less likely to locate matching donors. As such, appeals often come from racially minoritised patients. Prior research highlights the importance of narrative in health contexts, and donation in particular. However, the impact of stories on those telling them is underexplored. This article fills this gap, providing analysis of a range of interview, media and documentary data. It sketches out the contours of appeal work, showing how patients’ private lives become publicly exposed. It highlights how appeals might be understood as collective action on behalf of racially minoritised communities, flagging how those most affected by inequity often become central to the fight to redress it. Through this, the article extends an emergent ‘sociology of donation’, arguing for acknowledgement of media’s importance in contemporary donation contexts.

## INTRODUCTION

Next tonight, [a young child]…and his family were told last summer that he would need a stem cell donor in the next few years after he was diagnosed with a rare genetic disorder. But just a week ago his parents were told they now only have months to find a match. So they are asking anyone, particularly people of South Asian descent, to join the register, to give their son a fighting chance of survival.

(Televised transcript, appeal 1)

Individual narratives are employed around the world to recruit blood, organ and stem cell donors. These are thought to enable would‐be donors to imagine the benefactor of their decision to donate through centring the story of an individual. This strategy can recruit many donors and is seen as particularly effective in encouraging those thought less likely to enrol, like racially minoritised individuals whose lower engagement with donation links, for various reasons, to worse outcomes for racially minoritised patients (National BAME Transplant Alliance, 2012). However, whilst patient narratives are a well‐established element of recruitment, little attention has been paid to the experience of being the subject of such a narrative. What is it like for individuals whose stories are mobilised for this purpose? Furthermore, what might the implications be of donation systems’ reliance on individual narratives, particularly to mitigate existing inequities like those around race?

This article contributes to the emerging sociology of donation (in this journal, see Dimond et al. [2019]) by exploring these experiences, emphasising the important and entangled roles of narrative and media in the donation context. It argues that we must pay more attention to the experiences of those whose stories are used to increase participation in biomedical projects, including, but not limited to, tissue donor recruitment. This is because recruitment for such projects employs the work of individual patients and families, and this work is often distributed in ways that mirror existing health inequities, such that those most affected by inequity often become central to the fight to redress it.

## CONTEXTUALISING STEM CELL DONATION AND PATIENT APPEALS

Stem cell registries exist in 55 countries across six continents, comprising data of ∼40 million donors (World Marrow Donor Association [WMDA], 2021). These registries facilitate anonymous donation^1^ of genetically matched stem cells from donors to patients with haematological malignancies from blood cancers to sickle cell disease. The UK has four registries: two state‐run, and two charity‐run (Anthony Nolan and DKMS UK). Across Europe alone in 2019, 19,798 patients received transplants (Passweg et al., 2021). These are the individuals whose matches were found, but of global searches in 2020, 8% of patients found no match (WMDA, 2020).

Matches between donors and patients are thought to be more likely within ancestral populations, sometimes shorthanded as ‘racial’ or ‘ethnic’ groups (see Williams [2018] and Avera [2009] for critical analyses of these claims). In the UK, as across the global north, if you are white, your chances of locating a match are far greater than if you are not. This is because there have historically been disproportionately fewer racially minoritised registered donors, leading to fewer racially minoritised patients finding matches. Though there is not scope to offer an historical account of this, policy narratives suggest this is partly due to historical mistrust of health systems (Smith, 2018). In turn, the policy response has been to mobilise racially minoritised individuals and related organisations to encourage donation amongst their own communities. These individuals/organisations are often prompted into this work by their own experiences of loss, which they share through storytelling (see Williams, 2021).

Narratives go to the heart of this system. Indeed, the first registry was established in the 1970s after an appeal for a donor for a young boy who required a then‐experimental bone marrow transplant. His mother mobilised her son’s story with a view to locating his match, which produced a database of would‐be stem cell donors (Goldman, 2002). Today, registries around the world feature so‐called ‘patient appeals’ on their websites based around individuals currently unable to locate matches (e.g., Be the Match, 2021; DKMS, 2021). Many patients and families lead these appeals, hosting their own ‘donor drives’ (events where people can come to swab their cheeks, complete sign‐up forms and register as donors). These events are often coupled with traditional media coverage and social media activity to amplify appeal subjects’ stories in a bid that seeks—however improbably—to locate their own match.

These same appeals generate potentially vast swells of recruitment, increasing numbers of available donors for the broader patient population. Registries note appeals’ value particularly for addressing racial underrepresentation: ‘recruiting high numbers of potential donors from black, Asian and minority ethnic communities [is] thanks to high profile appeals for people with minority ethnic backgrounds’ (DKMS UK, 2018, p. 4). Registries also emphasise a strategic commitment ‘to identify grassroots patient appeals and support families wishing to share their stories’ (Anthony Nolan, 2016, p. 41). In the UK, a support structure within registries has developed to assist patients/families in running appeals, which is suggestive of their perceived vulnerability during often acute personal trauma, from being diagnosed with an illness for which treatment exists in concept (a transplantation) but not in practice (a matching donor).

The following provides the key conceptual contours of this article, focussing particularly on narrative, media and how they come to relate, with examples from both beyond and within the wider sociological literature on donation. Before this, however, it is useful to clarify terminology. Since the 1980s BME (‘Black and Minority Ethnic’) had been employed in the UK to describe people who are racially minoritised (i.e., not of the majority ‘White British’ or, in some cases, ‘White European’ population). BAME (‘Black, Asian and Minority Ethnic’), a more recent acronym, has precipitated much debate, whilst the term ‘ethnic minority’ is often framed as an inclusive one (see Aspinall, 2021). There is significant disagreement about these terms, and about what race and ethnicity gesture towards, the former associated with biological notions of difference, the latter a ‘cultural’ group that some suggest allows us to talk about race without using the word (see M’charek et al., 2014). With this in mind, this article adopts the term ‘racially minoritised’ to denote the processual, contextual nature of the notion of ‘minority’, preserving alternative terminology where present in data.

## NARRATIVES, MEDIA, DONATION

Narratives—the stories we tell and are told—are at the heart of illness experience, and there is a long tradition within the sociology of health and illness of exploring them. As Bury’s (1982) seminal intervention in this journal notes, narratives can be put to work, preserving sense of self in periods where illness causes ‘biographical disruption’; through sharing their stories with one another, as Charmaz put it, ‘people narrate their way into being’ (2002, p. 309). In donation contexts, we see these insights in action. Organ recipients find creative ways to wend anonymous donors into their own life stories (e.g., Sharp, 2006); donors, recipients and offspring of reproductive material enrol imaginaries of one another (e.g., Hudson [2020] on women’s accounts of their egg donors; Wheatley [2019] on offspring accounts of sperm donors).

Media allow narrative to circulate still further beyond our immediate social contexts; Seale noted in this journal nearly 20 years ago that analyses of health and media must converge, because ‘the greatest repository of stories in late modern societies is made up from the various organs of the mass media’ (2003, p. 514). Increasingly, we must add new digital media to this equation. Mobilising what Orgad describes as a ‘self‐story’ in a blog can help the person telling it make sense of their experience. Elsewhere, Vicari’s focus on Twitter discussions regarding the BRCA gene demonstrates how the material infrastructure of the platform allows individuals ‘to disclose—or not disclose’ (2020, p. 19) their personal connection to the risk‐raising gene, demonstrating the need to consider the role that media technologies themselves play in whether, how and with what consequence, narrative might be mobilised.

Implications of mobilised stories for subjects can be profound, too. In the context of medical ‘crowdfunding’, individual patient stories are shared widely across social media. This is not primarily to make sense of illness, but to encourage financial donations towards its treatment costs, requiring public exposure of personal health conditions/identity. In their powerfully titled article, *Better everyone should know our business than we lose our house*, Gonzales et al. (2018) explore the bind of feeling compelled to publicise private health‐related information about oneself with the hope that an audience will donate money. For their interviewees, financial need exceeded the discomfort of divulging personal health information.

Whilst experiences of crowdfunding have received significant attention, the experience of mobilising stories to provoke tissue, rather than monetary, donation, has received far less. This is despite their centrality in donor recruitment strategy. There stand to be important insights in focussing attention here, however, not least because critical media scholarship draws attention to how complex the relationship between media and narrative can be. Indeed, the subject of narrative—though potentially the initial teller of the story—cannot always control its sharing or reception. Whilst media attention might ‘convince broader publics’ of a given cause (Tufekci, 2013, p. 849), when messaging finds its way into mass media, those behind it have little sway in controlling a narrative (see Gitlin, 1980). Narratives can thus be transformed by intermediaries. Moreover, whilst *social* media may ‘appear to resolve the communication predicament’ (Poell & van Dijck, 2015, p. 528) for causes struggling to access *traditional* media, platforms’ technical infrastructures, from ‘retweets’ to proprietary algorithms, privilege content—and, thus, narratives—that are more favourable to platforms’ business models, offering an important material element to whose story ‘deserves’ to be seen.

What, then, of media in the donation context? Whilst narrative has been a perennial concern to sociologists concerned with donation, media has been less so. In the recent and important effort to draw together and establish a future agenda for the area, scholars acknowledge media’s role in how meaning is brought to ideas and practices of donation (e.g., Dimond et al., 2019), yet it is absent in a systems‐level framing of donation that incorporates within it clinicians and families, communities and cultures (Machin et al., 2020). This is perhaps because, whilst there is an extensive social science literature at the media/donation intersection that explores components of media in both how donation systems operate and how people come (not) to engage with them (Kim [2018] on Japanese press coverage of contaminated blood public health scandals; Kierans and Cooper [2011] on ethnicity‐targeted publicity campaigns for organ donors in the UK; Simpson [2011] on Sri Lankan blood donor advertising), less attention has been paid to how media itself, extensive and central to social life as it is, plays a vital role in the donation context. In the UK context alone, alongside patient appeals in the stem cell context, the UK’s NHS Blood and Transplant (NHSBT) actively seeks out ‘real‐life stories’ of successful transplant to ‘promote’ organ donation that can be shared on social media (NHSBT, 2021). Add to this digital media technologies like NHSBT’s appointment‐booking app, that ‘puts the power to save lives in the palm of your hand’ (Apple, 2022). These exemplify, alongside the patient appeals explored below, media’s multiple roles and increasing importance within donation systems.

Given the centrality of narratives in the sociology of donation, and their strategic value in recruitment practices for systems like stem cell donation, it is surprising that there is not more exploration of the experiences of the subjects of these stories. This inquiry is increasingly necessary because of the complex role that traditional and now social media play, in amplifying narrative and potentially complicating the role between a story’s initial teller, and the story told. The current intervention aims to explore these experiences, attending to how media are implicated in them. Underwriting this is an interest in the way the telling of these stories is often necessitated by existing racial inequity in the donor system, a point to which I return throughout the article, and in the discussion.

## METHODS

This article explores a selection of UK‐based appeals. In all appeals considered, patient racial identity figured in their narrative (e.g., ‘X has a rare tissue type because of his mixed heritage background’). Although accounts below might resonate with patients running appeals *regardless* of racial identity, the project from which this article stems explores recruitment of racially minoritised donors. It is probable that a disproportionate number of appeals feature racially minoritised patients (of 14 appeals live on one UK registry at the time of writing, 7—half—are of individuals described/identifying as other than white northern European [Anthony Nolan, 2021b]), justifying a focus on this experience, and what it tells us about racial inequities in donation contexts.

All appeals in the sample feature elements described earlier: *in‐person donor drives*, where potential donors can learn about donation, being told the story of the person requiring a match, oftentimes by the patient themselves. They are then invited to register as a donor, completing registration forms and swab kits provided by one of the UK registries to the appeal. Secondly, all incorporate *engagement with media*. This involves local, national or even international coverage on television, radio and/or newspaper often featuring interviews with patients and/or families, alongside details of how audiences can register (i.e., flagging upcoming donor drives, or links to registries’ online sign‐up forms). Whilst the earliest appeal pre‐dates social media, others established social media accounts across different platforms (see Table S1 for details).

Semi‐structured interviews were undertaken with individuals (*n* = 17). In addition to a group interview with three individuals working at a UK stem cell registry who had familiarity with the appeal process and their registry’s role in them, this figure comprises five patients and nine family members behind eight separate patient appeals (see Table S1 for further demographic detail).

Appeals were identified via observation of UK registries’ social media to locate appeals being shared. I was also aware of past appeals via my familiarity with the field. Appeals were then approached through direct contact via social media or email. Oftentimes, appeal families knew one another, and some appeals with which I had established relationships put me in contact with other appeals.

In some cases, patients at the centre of these appeals have died, either before the study began, or since the individual was interviewed. Even where this was not the case, anticipating interviews could elicit acute emotional responses, participants were invited to be interviewed as family groups. Where multiple participants are interviewed regarding one appeal, these were undertaken as group interviews. Most interviews took place over virtual platforms because of COVID‐19 restrictions, though two appeal interviews took place in early 2020. Interviews lasted between 1 and 2 h, and were professionally transcribed.

The article also incorporates analysis of media material (including social media and traditional media coverage) from three of these appeals. These data were collected through a mixture of a web scraping tool (Web Science Institute, 2020) and database searches (see Table S2 for a cross‐tabulation of the different kinds of data collected in this subsample). It also incorporates data from an analysis of grey literature (see Table S3) explored as part of the larger project to which this article is linked. Ethical approval was obtained from the author’s institution. Although some interviewees gave permission to be named, efforts have been made to anonymise all appeals/people in this article for consistency.

An abductive approach (Timmermans & Tavory, 2012) was employed for analysis, in which an ‘inferential process’ informed by familiarity with literature in the empirical area was adopted to theme interview talk. NVivo was used to facilitate this process. Data in media content were also analysed using NVivo, alongside interviews. Social media data underwent descriptive statistical analysis to measure frequency, and content or posts were read in Microsoft Excel and, where relevant, added to the NVivo outputs which collated themes from interviews/media.

## PATIENT APPEALS IN PRACTICE

This first section gives a sense of what patient appeals entail in practice. As noted above, patient appeals comprise two key components: *donor drives* generally run by those who are involved in the appeal, and *media activity* through a social media presence and/or traditional media coverage. By way of exploring these elements of appeals, this first section demonstrates the variety of effort that can go into creating and sustaining them.

The first appeal considered here came about because the woman at the centre of it, who describes herself as Indian, could not find a matching donor for her transplant. She made appearances on national television and radio and wrote an op‐ed in a national newspaper, offering watchers, readers and listeners versions of a narrative that shared her diagnosis, the disruption it caused her and her family, as well as her prognosis. She also established a Facebook page which shared the narrative, both through posts of updates in the story, and by listing donor drives where people could come and hear her tell the story in person, and register as donors. In our interview, she emphasises the network of support that solidified to help her run donor drives, as volunteers offered help with them:

… people just kept coming out of nowhere to help me [at events], and I just thought it was amazing that they just wanted to give up their time, or their energy…to try and push things along, and that in itself gave me a lot of hope, and I got a lot back from that….

She also acknowledged that these same events brought significant challenge with them:

[W]hen we were doing the…[events, audiences would] realise…“…I’m actually speaking to the person that we’re trying to save her life”, and they would go, “I’ve brought my whole family to come and register”, and that’s the momentum that you need…[Soon] I did start to feel really exhausted…everyone kept saying “slow down…you don’t need to go to every single thing”. But it’s really hard to let go…when you feel like, if I’m there in person, it will have more impact…I was really worried about germs and…my consultant didn’t want me to go to too many [events] because of the exposure…[E]ven having media interviews and doing all of that…does take energy out of you…I ended up going into hospital for almost three months…because I was going too full‐on with the campaigning…but what do you do?

(appeal 2, patient)

She, like other participants, saw value in turning up at donor drives, providing the ‘momentum’ needed to personalise a story, in effect putting her illness identity (Charmaz, 1995) to work as the ‘person’ at the heart of the story being told, whose life donors are ‘trying to save’. Yet her account of hospitalisation speaks to the challenge of overlaying an active appeal schedule—events and media appearances—with treatment. Blood cancer patients are often heavily immunocompromised, and advised to recuperate from treatment, maintaining physical distance from others. Registry workers interviewed for this study expressed sometimes trying to discourage individuals from appeals if the patient was felt to be especially vulnerable. However, this patient sees her presence as key to generating registrations. Moreover, there is an evident sense in her rhetorical ‘but what do you do?’ that, despite objections from those around here, she had little choice in the matter.

This perhaps shines a slightly different light on what the patient describes as the sense of ‘hope’ she gained from her appeal work. This might be a perception that she was improving her own odds of finding a match; however, the need to improve these odds itself is telling of something more structural—an existing dearth of suitable donors. In other words, even though this work might generate ‘hope’, the initial need for that hope is itself contingent on this lack.

Consider the account of another woman who was frequently in hospital during appeal 3. Identifying as mixed race, she was advised that her match was likely to be found from a donor of a similar ancestral heritage to her own but that there was no matching donor for her on the registry. Her family and friends ran donor drives, and she featured in national and international news outlets. Her story—via quotes from interviews with her and her family—was featured in articles from her local newspaper, to one of the US’s most widely circulated national daily papers. Her appeal’s social media activity, including images of her mid‐treatment, was also heavily circulated online. Posts garnered Retweets from celebrity footballers, comedians and authors. In an interview, she notes how:

you feel a bit powerless and all you have to do is just wait and find out whether you’ve got a match or not. And the wait was quite long. [Appeal activity] kept me really busy and kept my mind off things in the meantime, just having stuff to do…[T]here was a point where I just spent all day responding to messages…[and] I’d never realised how important keeping producing content is…so that it always comes up on people’s newsfeed, there’s a new thing to click on…[N]ow, because it’s so inactive, literally 100 people may see it compared to the thousands of people…seeing it before. And it’s only because of those algorithms and everything, you need to just keep posting content and people are seeing it. And so that’s why we just came up with different ideas of another thing to post…

(appeal 3, patient)

**FIGURE 1:**
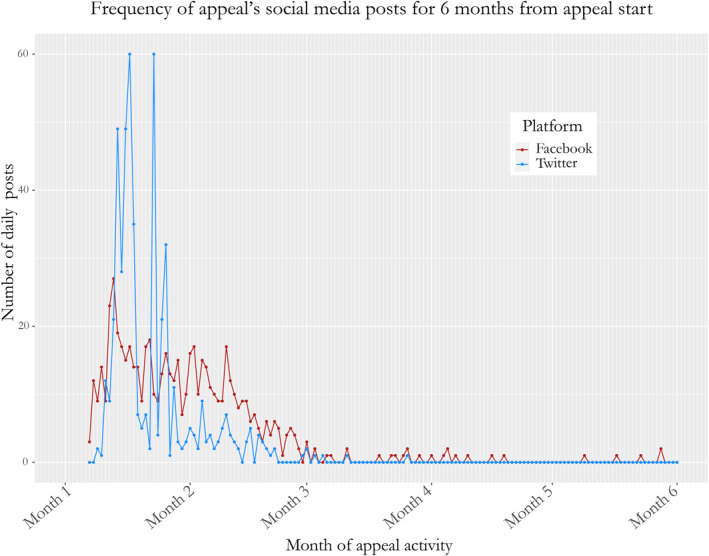
Frequency plot of posts from appeal 3’s social media over 6 months

During the 12 months in which appeal 3 was active, its Facebook pages (it had multiple pages in different languages, maintained by her, some close friends and family) posted 635 times. Of these, 583 were made in the 2 months from the appeal’s start, averaging nearly 10 daily posts. On Twitter, via two accounts in separate languages, 505 Tweets were posted over the 12 months, 502 of which were sent in that first 2‐month period, averaging around 8 daily Tweets (see Figure 1).

Like the patient in appeal 2 above her who felt compelled to attend events to sustain momentum, the patient in appeal 3 recounts the time‐consuming nature of social media maintenance. After Vicari (2020), then, considering social media platforms’ roles in how narrative’s mobilisation is experienced is key. Alongside responding to messages (interviewees noted receiving direct messages from well‐wishers, spammers and queries about how to register), the patient in appeal 3 describes the ‘need to just keep posting’ to retain visibility; she generated content including treatment updates along with pictures. Posts also included stories of people signing up because of the appeal, and stories of other patient appeals. These posts serve to add detail and texture to the narrative this appeal is sharing, and represent the significant effort of its telling. This is a point emphasised by Kenworthy’s (2019, p. 6) analysis of crowdfunding wherein already‐trending campaigns draw potentially exponential attention, whilst those with less engagement are rendered algorithmically irrelevant. The ‘threat of invisibility’ as media scholar Taina Bucher locates in Facebook’s newsfeed algorithm (2012) is clear here, as those behind the appeal try to ensure it remains in people’s purview.

As such, we might give pause to the fact the appeal occupied her attention—which she describes as a means of distraction from the sense of ‘powerlessness’ she felt in hospital. Like the ‘hope’ described by the preceding account, the need to preoccupy herself in the first place is contingent on a wait (indeed, ‘all you have to do is just wait’) bypassed by those who *do* find matches. As Petersen and Wilkinson (2015, p. 116) note in their consideration of hope’s role in the context of health, whilst ‘hope may be personally meaningful—indeed, embraced as “empowering” […these] individual biographical experiences are inextricably linked to wider socio‐cultural and historical contexts and processes’. In this sense, the ostensible ‘positives’ of appeal work—framed in terms of generating hope or empowerment—are essentially filling gaps (an absence of hope, or long stretches of waiting) that, were matches more readily available, would not exist.

Data here are presented to give a sense, through participant accounts, of what an appeal can entail. They surface how appeals require work, involving patients’ resources—their bodies, energy, time. Whilst these examples also demonstrate that appeals could foster a sense of hope or shared experience with family or friends, and offer distraction in distressing times, it is also important to remember that this need for hope and distraction emerges out of a wider deficit in the registry of suitable donors for all the patients who need them.

## THE PRIVATE, MADE PUBLIC: VISIBILITY AS A NECESSARY COST OF APPEAL WORK

Appeals are built upon the narratives of patients and their families. Telling stories at donor drives is coupled with media engagement which entails sharing details of a patient’s diagnosis, prognosis and/or treatment (consider the epigraph at the start of this article, for example). This section explores how that visibility—so sought‐after by appeals, mirroring activist media strategies (see Tufekci, 2013)—itself carries a cost, as private lives and the stories about them are rendered public.

Registry workers interviewed were cognisant of the challenge that engaging with media might present appeals:

it’s quite a big thing, isn’t it, to put your story out there, front and centre?…[O]nce we have a press release which is essentially their story, written in the style of a news story, the family will sign that off…we know journalists…and we’ll essentially pitch that to them…and make sure that we are telling the story in as emotive and impactful way as possible, so that it’s…spotted and…we’re then able to make it from a press release into their publication or onto their broadcast or news outlet…[O]nce a press release is out there, we don’t have control over how exactly it lands.

(registry worker 1)

Registries often provide a through‐line to media, issuing press releases on behalf of appeals. Noting their effort to generate emotion through storytelling (see Williams, 2021), this registry worker acknowledges the weight that a story’s potential visibility carries with it. It is ‘a big thing’ over which ‘control’ is ultimately lost.

A striking example of this struggle to maintain ‘control’ comes from appeal 4 which was based around a young girl requiring a matching donor for her transplant. Described by her family as having a mixed heritage, the reason she was unable to locate a match was understood to be because the registry lacked donors of a similar mixed heritage to her own. In this appeal, her family sought media exposure themselves by activating connections in their own social networks. They established a website and social media accounts in the appeal’s name, running donor drives in their local area. Through all of this, the appeal generated significant media attention. During an interview with her father and aunt, they describe the global media appetite for the story:

ITV [a UK‐based national television channel] came to the hospital, filmed…a two‐minute piece on [her], and then things started to snowball…[the registry] said 12,000 [registered…] in the first ten days [and they were] used to getting about 30 a day, so this was like, “oh, okay, bing, something’s working”…We were getting write‐ups in 13 different countries about this. There were articles all over the place…[We] were on Australian TV. The [Australian registry was] inundated [by people] registering…[T]he system was trying to keep up…We had donor drives then in Singapore and all over the place, and we were on the New Zealand bone marrow register[’s]…front page for weeks.

(appeal 4, father)

As he notes, media attention generated massive donor registration spikes with which registries struggled to ‘keep up’, reasserting appeals’ strategic value for recruitment. As part of his daughter’s appeal, he also maintained a blog to refer to in conversations with doctors, and to keep family apprised. They soon realised the password‐protected blog had been more widely shared, and was receiving unexpected visitors. In discussing this, their account adds texture to the experience of being at the centre of media attention:

Father: Happy photos and good moments were less interesting than bad moments…[O]n the day [my daughter] died…50,000 people…looked at that webpage…[T]hat was the biggest spike we’d seen…Horrific stories [of her treatment as] we were being funnelled…towards palliative care…and all of that meant more viewing. It made good copy…[It] felt…a bit like a TV series, albeit a literal one, that people were…obsessed by this and going in multiple times a day, more than me, to check on the next bit.

Aunt: …It was like a car crash feeling…[W]hen she died it made the press.

Father: I said [to the journalist], it’s just so helpful that you’re supporting. He said, to be honest…every time we write about [your daughter], we get all these likes, inundated. So there was a reason for it, and it was selling newspapers.

In this account, the blog became a site of exposure to—even ‘obsession’ with—the girl’s story (indeed, there is perhaps a currency in young age that potentially appeals to a broader audience of spectators [see Seale, 2002]). But though coverage may have extended the appeal’s impact on donor registrations, her family are attuned to the economic value of their decision to share her story—rendered here as ‘good copy’ for ‘selling newspapers’.

The equation of the ‘horrific’ with increased attention speaks to how appeals themselves might become a kind of media spectacle. As media scholar Chouliaraki (2006) notes, the relationship between media representation and audience action is complex. Large audiences do not guarantee corresponding uptake of the invite to register (nor, as we will see shortly, does uptake guarantee the named patient will locate their own match). Indeed, at times the family behind appeal 4 infer her story was consumed as much as entertainment—‘like a TV series’—as an appeal to action. As such, the father and aunt must reconcile their decision to share the girl’s story to find her match, with the appetite of the wider media system in which the story gained traction.

Though most appeals explored here did not garner such international attention, patient perspectives gathered suggest that even local or national attention could bring personal discomfort to the individuals featured, reasserting the registry worker’s point that sharing a story can be ‘a big thing’. In the case of a man who began appeal 5 to locate his own donor, his most prominent exposure was on television and in newspapers, which were used to advertise the donor drives he was running. Self‐identifying as British Asian, he too had been unable to find a matching donor on the registry for the transplant that would treat him. For him, realisation of his appeal’s reach was from seeing himself on television. It was met with unease:

I like to keep my anonymity, and…keep my private life private. So, I found that a really uncomfortable experience, but it’s one I had to get over[…One day,] the nurse was actually giving me…medication…And we had a TV in there and my face came up on the TV and we both looked at each other, and…I found it uncomfortable…she was over the moon with it…but I didn’t share that…

(appeal 5, patient)

As with the woman in appeal 2 who felt no choice but to attend donor drives to improve her appeal’s impact, this man felt that he ‘had to get over’ his discomfort with his private life’s exposure—exposure he himself sought to widen the potential audience of his appeal.

In a context where an appeal might feature in local or national traditional media but also travel across social media platforms in unpredictable ways, these dynamics of compulsion and discomfort could arguably be amplified. Below is an excerpt from an interview with a woman at the centre of an appeal whilst a teenager. Identifying as Indian, she and her family had been told that the registry was unable to provide the matching donor required for her transplant to go ahead. Her appeal to locate a match featured donor drives in her local area, many of which she attended. Her family also established social media accounts populated with multiple images of her, edited to feature a textual account of her diagnosis and details of how people could register as donors, along with a hashtag featuring her name and links to her appeal’s Twitter, Facebook and Instagram pages:

I’m a very private person, I don’t have any social media…so me having my face plastered everywhere…[my] dentist would recognise me, and everywhere I’d go somebody…was like, “oh I saw your thing on Facebook”…[M]y family [were] going crazy…posting it everywhere. So, for me personally I felt really horrible…I hated it because I was going through a really rubbish time in my life, I didn’t look great, I felt really chubby and I didn’t want to have that attention…there’s a time in your life when you want to hibernate, and I couldn’t…[D]uring that time I thought okay, [my father’s] going to all of these fairs and trying to get people to join up, so I’ll help…and be the poster child there and try and get people to join up…it’s all written in stone now. So, if anybody future looks me up it’s just going to come up with all [the appeal material].

(appeal 6, patient)

Like the patient in appeal 5 above her who articulated discomfort with his television coverage, the young woman’s account suggests that appeal attention via social media was similarly uncomfortable. This was compounded by social media’s capacity to generate an enduring imprint of online activity (see Mayer‐Schönberger, 2011). This allows stories to gather fixity, notes Orgad of self‐stories shared online, as they ‘transcend the boundaries of a patient’s private sphere…[becoming] publicly available “property” to which the storyteller is committed’ (2005, p. 149). This means an appeal (and associated disclosures of illness) might be ‘written in stone’, enduring beyond its active period, with little control over who might see it.

Importantly, unlike the man in appeal 5 who led his own appeal, appeal 6 was driven in significant part by the patient’s family. This is not uncommon in this sample, often with younger patients’ families playing the most active role in appeal work. She wanted to ‘hibernate’, retract from public life, during her illness. Yet she ‘couldn’t’, instead feeling compelled to attend events, echoing the compulsion articulated by other participants above her. However, her sense of exposure—from her family’s ‘crazy’ amounts of social media sharing to being the ‘poster child’ for her father’s efforts at donor drives—is entangled in her position within her family, whose concern for her and desire to locate her match is articulated in appeal energy. Her account is indicative of how these appeals, though ostensibly about individuals, are often situated within familial networks. Donation scholars acknowledge how donation comprises an assemblage, including ‘a tangled web of people’ including both the donor and their family (Machin et al., 2020, p. 6). Appeals demonstrate how this assemblage extends to would‐be recipients and their own families, whose interests lie in their loved one locating a donor.

## ‘IMPROVING THE ODDS FOR EVERYBODY’: INDIVIDUAL WORK FOR COLLECTIVE BENEFIT

Thus far, we have seen how appeal work takes significant effort, and can generate an uncomfortable level of visibility for those involved in it. Importantly, whilst appeals may aspire to locate their subject’s match, registrations might benefit *any* future patient. If registrants prompted by appeals eventually donate, the recipient is unlikely to be the original appeal subject, given the specific genetic matching required. Thus, appeals require time and energy, often‐uncomfortable levels of public visibility (sometimes in perpetuity), all whilst not necessarily locating the subject’s match. Indeed, appeals can actively construct the subject as one amongst many (often racially minoritised) potential beneficiaries, allowing us to understanding appeals as a mode of collective action.

The next account comes from appeal 7, which included both international and local traditional media coverage and social media activity, including a website and blog, all led by the man around which the appeal centred. Told by his clinical team that his specific mixed heritage background was the reason why a donor with his rare tissue type was not found on the registry, he sought to establish an appeal. His website features a pair of numbers flanking a photograph of him looking forwards holding a megaphone as if shouting about registration. One number is total donors registered via his work (at interview, ∼45,000; currently ∼90,000). The other is the amount of those who have gone on to donate to somebody (at interview, 15; currently 17). In our interview, I ask about those other beneficiaries. He says:

I probably won’t find a match…because my genetic heritage is so diverse and rare…there just aren’t that many people out there…But I also see the value and benefit for other people. So I’ve…disassociated myself from being core to all of this…I am happy doing this for other people, because we’ve got 15 examples of it working…out of 45,000 [donors]…I’ve made peace with my odds, and I’ll continue to work to try and break them, but because it’s working we’re improving the odds for everybody and it means that hopefully they won’t have to face [my situation].

(appeal 7, patient)

He concludes he is unlikely to locate a match because of his heritage, so his appeal focuses on trying to ‘improve the odds for everybody’. The conviction that others should not face the lack he was confronted with speaks to the entanglement of individual need with a wider *collective* need. Whilst his mixed heritage background has led to some targeted media coverage in places with which he shares his diasporic affiliation, his focus is relatively generalised with a key gambit in his appeal about increasing the general number of donors on the register.

Consider this in conversation with an extract from an interview with a father who went on to establish a Black donor‐focussed recruitment charity after he and his partner ran appeal 8 in the late 1990s to locate their child’s match. Like the other appeals, the family had been told that a matching donor for him could not be found on the registry. It had been explained that this was because of a dearth of Black donors:

everything that we do…is linked to leaving a strong legacy for future generations…[It] started in January [late 1990s] when we were told…[our son] needed a transplant, and I [thought] there’s a baton on the floor…because a father was sitting here, a mother was sitting there, and they didn’t pick up the baton because they realised: this is a long marathon we’re going to run and I don’t think we can do it…[I]t’s a case of…we’ve left you a legacy, you carry on the work.

(appeal 8, father)

Again emphasising the role patients’ surrounding families often play in appeals, as in appeal 6 above, this father describes picking up a metaphorical baton—taking on responsibility for increasing donorship. It is suggestive of the complexity of both a familial sense of duty, and the broader notion of community, too: others confronted with the same inequity did not, or could not, pick up that baton for their own child. The ‘long marathon’ of this kind of appeal work evokes how appeals can, as we have seen above, be hard work—perhaps too exhausting or exposing for some.

Whilst at the time of the appeal the son’s own donor was sought, he eventually died and his parents continued. The baton, moved forward as their ‘legacy’, might now be furthered by ‘future generations’, evoking an innumerable future collective. Not mentioned in this extract, but visible in the charity’s work, is the centrality of race. The organisation’s purpose is explicitly racialised, promoting awareness of donation in the Black and mixed heritage community. Importantly, their child’s eventual donor (whose donation provided several years of extended life for him) was from America, far from their UK‐based appeal work, but many of those whom they recruited in their son’s name have gone on to donate for others.

Similarly, appeal 3 has a noticeably racialised remit latching onto this sense of collective racialised benefit. Demonstrating similar commitment to expanding matching chances beyond the patient’s own, it notes the plurality of potential beneficiaries through ‘diversifying’ donorship: across its social media and website, the appeal is described as searching ‘to save lives’ and ‘diversify the bone marrow registry’. Furthermore, the appeal website features stories of other racially minoritised patients currently seeking matches, with a grid of patients’ pictures which link to their own appeal webpages.

One UK registry notes in its annual report that the year when appeal 3 was active:

saw a huge surge in people from black, Asian and minority ethnic (BAME) backgrounds joining…in response to inspiring patient appeals…40% of people who applied online during these campaigns were from BAME backgrounds, compared to the average rate of 14%.

It also flags that around 50,000 people, of whom half identified as ethnic minority, ultimately signed up from the appeal. These people now stand to be anybody’s match, rather than the match of the individual whose story engaged them.

The multiplicity of potential beneficiaries—an appeal’s essentially *collective* nature—as a motivating force, shares qualities with health activism. In Brown et al.’s field‐defining article on health movements, they note that a central element is ‘the emergence of a collective identity as a mobilising force’ (2004, p. 55). The ‘collective’ of much of this work is often a group with a specific condition. A group can also form around a social location; a constituency‐based health movement aims to address ‘disproportionate outcomes and oversight by the scientific community’ (Brown et al., 2004, p. 53). For Brown et al., women’s or queer health movements are examples. However, examples also exist in the context of race (see Alondra Nelson’s [2011] work on the Black Panther Party’s health activism, or Melissa Creary [2018] on Brazilian sickle cell disease organising). Even though an individual might be the subject in a narrative, appeals—like other kinds of health activism—stand to have a wider set of beneficiaries. It is vital to acknowledge this, as it allows us to better grasp the complex role that appeals play in the broader context of racial inequity in stem cell provision, a point I return to below.

## DISCUSSION AND CONCLUSION

Sociological studies of donation have given significant attention to the role of narrative, as donors, recipients and kin find and bring meaning to their experiences of donation. Narrative’s use extends well beyond this too, as stories of patients in need are mobilised to engage new stem cell donors. These stories do things (Bury, 1982; Charmaz, 2002); here, compelling registrations. But they do more: they have implications for the people who tell them. We cannot understand these implications without also taking seriously the role of media. Indeed, this article seeks to demonstrate the importance of media in the context of donation. Media, I want to suggest here, is vital to donation systems, and will become only more important to them as new media technologies are enrolled in everything from donor recruitment to recipients informally seeking their anonymous donors online. Taking the importance of both narrative and media as a departure point, the current article has thus sought to explore the experience of those whose narratives are mobilised—and often shared widely with and through media—to recruit stem cell donors. Returning to the initial provocation, then, what are the implications of appeal work for the individuals behind them?

In the context of stem cell donor recruitment, where the telling of a narrative emerges from a lack, stories may be challenging to tell, requiring significant energy on behalf of the teller. They are also told in a media‐rich context where traditional media, and increasingly social media, become the vehicle for attention. This vehicle runs on the efforts of appeals to keep posting, but is also predicated on the decision to make a story visible.

Perhaps central here is asking who decides to make the story visible? Journalists must see value in a story to share it; social media platforms’ material infrastructures demand extra detail in order to push an appeal up an algorithm and into public view. These effectively take decision‐making on *how* a story is (not) shared out of the hands of a given appeal. But even within an appeal, it is not just patients, but also their families, who put in much of this effort. Family effort is no doubt underwritten by a profound amount of care, ignited by a fear of loss of their loved one. This casts further light on the network of actors that make up the donation assemblage (Machin et al., 2020), but also centres us on an important point: that appeals are things that those involved in them evidently feel compelled to do specifically because they or a loved one cannot find a match.

In short, it perhaps is not appropriate to describe the undertaking of an appeal as a ‘decision’ at all. The woman whose consultant told her to rest, ‘but what do you do?’; the teenager who wanted to ‘hibernate’ but who felt she ‘couldn’t’ because her family needed her help with the appeal; the man who flinched at seeing himself on the TV but ‘had to get over’ it. Considered in the context of Alondra Nelson’s analysis of Black health activism, these people, faced with ‘health crises that disproportionately affected them’ as racially minoritised people, ‘had little choice but to provide their own solutions to what ailed them’ (2011, p. 26).

Indeed, this work is not only—or even mainly—for the subject’s benefit; for the man who generated 17 matches for others but not himself, recruiting donors ultimately helped unknown strangers. As such, individual exposure has collective benefit. But an enduring racial inequity in access to various kinds of donated tissue necessarily underwrites this study. The stem cell inventory is a resource that has historically underserved patients from racially minoritised backgrounds, in large part because recruitment mainly penetrated middle‐class white communities (see Brown et al., 2011), failing to engage racially minoritised groups. Though this has changed in recent decades, the system remains inequitable. Whilst most white patients who need one will find a donor, moving to transplant and recovery, their illness remaining a private matter, racially minoritised patients continue to face worse odds. Thus, appeals which emerge when a match is *not* found disproportionately regard racially minoritised people.

In the context of health, it is often patient communities who—feeling disregarded or underserved—place energy into their own improvement. A rich body of work flags moments where states or institutions have under‐resourced or divested themselves of responsibility to address certain citizen groups’ concerns, and communities have stepped up (see Creary, 2018; Epstein, 1996; Nelson, 2011). Patient appeals, which mobilise personal narratives, and attempt to amplify them far and wide, can share consonances with these efforts. It is important to note, however, that in the case of stem cell provision, there has *not* been wholesale divestment. Over the past two decades, a clear policy priority has been to improve provision to all who need it. Frequent reference is made in UK policy to improving access. As part of this, appeals have become to some extent institutionalised (e.g., in the form of registries providing support to amplify appeals). In this sense, institutions effectively buttress appeals. This is because those working in these systems recognise the clear efficacy of such narratives to communicate the magnitude of suffering and compel action, particularly to a racially minoritised audience, whom they have evidenced a commitment to trying to recruit as part of a broader push towards health equity.

All of this is not to suggest that narrative is something we ought to move away from in the recruitment context. Indeed, extrication would be impossible as narratives are central in social life, which is perhaps why they are so effective for recruitment. Nor can we discount the value that some patients draw from appeal work. Hope and distraction, though contingent on a lack, may well bring reprieve in difficult times. These are their experiences, their stories to share as they choose (though patients do not always feel they have a choice).

If telling stories can take a toll, and if this effort is distributed disproportionately to racially minoritised communities, it is incumbent upon us to reflect on the role of the patient appeal—not just in the context of stem cell donation but in related systems like blood and organ. Ensuring that emotional support is provided to patient appeals—something recently acknowledged in policy (APPG on Stem Cell Transplantation, 2021) is obviously crucial, but what else is missing such that patients take on this work themselves? How might resource be channelled into other forms of recruitment and outreach work? For instance, if policy acknowledges that community organisations have the experience to undertake effective recruitment in local, targeted ways that do not require significant media attention on individual stories, then perhaps more financial support needs to find its way directly into the hands of such groups. Perhaps then, eventually, fewer patients and families will feel compelled to share their personal stories, to improve the odds not just for themselves, but for the racially minoritised communities they effectively come to represent.

## AUTHOR CONTRIBUTION

**Ros Williams:** conceptualisation, formal analysis, funding acquisition, investigation, methodology, writing—original draft preparation, writing—review and editing.

## Supporting information

Supplementary Material S1

## Data Availability

The data that support the findings of this study are not publicly available due to privacy or ethical restrictions, and risk of identification to anonymous participants.

## ENDNOTE

^1^
Thanks to an anonymous reviewer for noting UK recipients are invited to write to their donors (see Anthony Nolan, 2021a), and for flagging accounts of recipients independently seeking their donors via social media.
