# Supplementary material for: ‘Improving the odds for everybody’: Narrative and media in stem cell donor recruitment patient appeals, and the work to redress racial inequity
Source: Sociol Health Illn. 2022 Aug 5;44(7):1114–31. doi: 10.1111/1467-9566.13505 (PMC9545486; doi:10.1111/1467-9566.13505)
Supplement: Supplementary file 1 — Supplementary Material S1 [file SHIL-44-1114-s001.docx]

## **Supplementary Material**

### **Table 1 Appeal details and data included in study**

| **Appeal identifier** | **Patient descriptor at time of appeal^1^** | **Period of appeal^2^** | **Traditional media coverage^3^** | **Social media presence at time of appeal** | **Media analysed for study (see Table 3)** | **Patient interviewed^4^** | **Family members / Friends interviewed?** |
| --- | --- | --- | --- | --- | --- | --- | --- |
| **Appeal 1** | Boy, South Asian | Ongoing, beginning 2019 | Scale: Local, national (UK), national (Indian), ethnicity-specific outlets; Types: radio, television, newspaper | Own website, Facebook, Twitter, Instagram | X |  | Mother, Father |
| **Appeal 2** | Woman, Indian | A year during 2015-2020. Patient continues to appeal for donors for others. | Scale: Local, national (UK), ethnicity-specific outlets; Types: radio, television, newspaper | Facebook, Twitter |  | X | None |
| **Appeal 3** | Woman, mixed race/mixed heritage | 4 months during 2015-2020 | Scale: Local, national (UK), national (inc. North America, Australia, Europe, Asia), ethnicity-specific outlets; Types: radio, television, newspaper | Own website, Facebook, Twitter, Instagram, YouTube | X | X | Mother, Father, Brother |
| **Appeal 4** | Girl, mixed raced/mixed heritage | A year during 2010-2015. Family created ongoing charity | Scale: Local, national (UK), national (inc. USA, Asian countries, European countries), ethnicity-specific outlets; Types: radio, television, newspaper | Own website, Facebook, Twitter, Instagram, YouTube |  |  | Father, Aunt |
| **Appeal 5** | Man, British Asian | 2 years during 2010-2015. Patient created ongoing charity. | Scale: Local, national (UK); Types: radio, television, newspaper | Own website |  | X | None |
| **Appeal 6** | Teenage girl, Indian | 1 month during 2015-2020 | Scale: ethnicity-specific outlets; Types: newspaper | Facebook |  | X | Father |
| **Appeal 7** | Man, mixed heritage | Ongoing, beginning 2018 | Scale: Local, national (UK), national (inc. Macau, Australia); Types: radio, television, newspaper | Own website, Facebook, Twitter, Instagram, YouTube | X | X | None |
| **Appeal 8** | Teenage boy, Black British | During 1995-2001. Family created ongoing charity | Scale of coverage: Local, national (UK), national (USA), ethnicity-specific outlets; Types of coverage: radio, television, newspaper | Appeal pre-dates social media |  |  | Father |
| **Total:** | | | | | | **5 patients** | **9 family members** |
| ^1^ Ethnic/racial identity is taken from self-definition by the appeal in their own public-facing activity. To minimise risk of identification, details of age and diagnosis are withheld.  ^2^ To minimise risk of identification, dates are kept vague. Where an appeal is no longer running, a patient might have found a match or have passed away. In certain instances, patients or their families have continued to appeal, sometimes attaining charitable status.  ^3^ Ethnicity-specific outlets refer to journalistic enterprises that target a specifically racialised audience (e.g., BBC Asian Network, Black-British Newspaper ‘The Voice’)  ^4^ Where patients were not interviewed, this was because patients (appeals 5 and 8) had died and their families were interviewed about their previous experience with the given appeal. In one case, the parents of the child in appeal 1 who was below the age of ten were interviewed without him present because of his age. | | | | | | | |

## **Table 2 Media data relating to appeals**

| **Appeal identifier** | **Period of data collection^1^** | **Tweets mentioning appeal^1^** | **Facebook posts produced by campaign^2^** | **YouTube videos produced by appeal (cumulative duration)^3^** | **Blog posts published on appeal website** | **Print media articles discussing appeal^4^** | **Radio and television articles discussing appeal (cumulative duration)^5^** |
| --- | --- | --- | --- | --- | --- | --- | --- |
| **Appeal 1** | October 2020 | 214 | 280 | 9 (1hr29m) | No blog | 22 | 6 (30mins) |
| **Appeal 4** | February 2020 | 6,513 | 635 | 37 (51m) | 29 | 109 | 18 (1hr11mins) |
| **Appeal 7** | June 2020 | 915 | 73 | 16 (1hr51m) | 65 | 76 | 24 (2hrs37mins) |
| ^1^Tweets were scraped through WebDataRA (Web Science Institute 2020), which is limited to data that is visible to users on the Twitter website. It is therefore possible that some tweets were not captured via this method. Tweets captured were all individually read. Where tweets were non-English language, they were counted to give frequencies, but not read as part of analysis.  ^2^Posts published on the campaigns’ Facebook pages were scraped with WebDataRA and individually read. Where posts were non-English language, they were counted to give frequencies, but not read as part of analysis.  ^3^Videos published on the campaigns’ YouTube pages were individually downloaded, watched and transcribed. Where videos were predominantly non-English language, they were excluded.  ^4^Print media were collected through Nexis and Google news searches using relevant search terms (campaign names and central individual’s name). Only English language news sources were analysed. Duplicates were removed, including syndicated newspaper content which was included only once where published in multiple local newspapers.  ^5^Radio and television coverage were located through URLs shared via campaigns’ social media and this was supplemented through Nexis searches and Box of Broadcast requests. | | | | | | | |

## **Table 3 Grey literature analysed**

| **Year** | **Authoring Body** | **Title** |
| --- | --- | --- |
| 2010 | Stem Cell Strategic Forum | The Future of Unrelated Donor Stem Cell Transplantation in the UK: Part 1 Findings and Recommendations |
| 2010 | Stem Cell Strategic Form | The Future of Unrelated Donor Stem Cell Transplantation in the UK: Part 2 Annexes |
| 2012 | National BAME Transplant Alliance | A Voice for Change, A Vision for the Future: Strategic Plan 2012-13 to 2014-15 |
| 2014 | Anthony Nolan | Annual Report 2013/14 |
| 2014 | DKMS UK | Annual Report 2014 |
| 2014 | Anthony Nolan | 40 Years of Life Saving |
| 2014 | Anthony Nolan | State of the Registry 2014 |
| 2015 | Anthony Nolan | Annual Report 2014/15 |
| 2015 | DKMS UK | Annual Report 2015 |
| 2015 | Anthony Nolan | State of the Registry 2015 |
| 2015 | Stem Cell Oversight Committee | Unrelated Donor Stem Cell Transplantation in the UK: Effective Affordable Sustainable |
| 2016 | Anthony Nolan | Annual Report 2015/16 |
| 2016 | DKMS UK | Annual Report 2016 |
| 2016 | Anthony Nolan | Lifesaving Science: Anthony Nolan's Research Strategy |
| 2016 | Anthony Nolan | State of the Registry 2015 |
| 2017 | Anthony Nolan | Annual Report 2016/17 |
| 2017 | DKMS UK | Annual Report 2017 |
| 2017 | Anthony Nolan | State of the Registry 2016 |
| 2018 | Anthony Nolan | Annual Report 2017/18 |
| 2018 | DKMS UK | Annual Report 2018 |
| 2018 | Eleanor Smith, MP (Chair of review) | Ending the Silent Crisis: A Review into Black, Asian, Mixed Race and Minority Ethnic Blood, Stem Cell and Organ Donation |
| 2019 | Anthony Nolan | Annual Report 2018/19 |
| 2019 | Anthony Nolan | State of the Registry 2018/19 |
